# Threats from and Countermeasures for Unmanned Aerial and Underwater Vehicles

**DOI:** 10.3390/s22103896

**Published:** 2022-05-20

**Authors:** Wahab Khawaja, Vasilii Semkin, Naeem Iqbal Ratyal, Qasim Yaqoob, Jibran Gul, Ismail Guvenc

**Affiliations:** 1 Computer Systems Engineering Department, Mirpur University of Science and Technology (MUST), Mirpur 10250, AJK, Pakistan; wahab.ali@must.edu.pk (W.K.); naeemratyal@must.edu.pk (N.I.R.); qasim.cse@must.edu.pk (Q.Y.); jibran.cse@must.edu.pk (J.G.); 2 VTT Technical Research Centre of Finland, Tietotie 3, 02150 Espoo, Finland; vasilii.semkin@vtt.fi; 3 Electrical and Computer Engineering Department, North Carolina State University, Raleigh, NC 27606, USA

**Keywords:** classification, countermeasures, detection, tracking, unmanned aerial vehicles (UAVs), unmanned underwater vehicles (UUVs)

## Abstract

The use of unmanned aerial vehicles (UAVs) for different applications has increased tremendously during the past decade. The small size, high maneuverability, ability to fly at predetermined coordinates, simple construction, and affordable price have made UAVs a popular choice for diverse aerial applications. However, the small size and the ability to fly close to the terrain make the detection and tracking of UAVs challenging. Similarly, unmanned underwater vehicles (UUVs) have revolutionized underwater operations. UUVs can accomplish numerous tasks that were not possible with manned underwater vehicles. In this survey paper, we provide features and capabilities expected from current and future UAVs and UUVs, and review potential challenges and threats due to use of such UAVs/UUVs. We also overview the countermeasures against such threats, including approaches for the detection, tracking, and classification of UAVs and UUVs.

## 1. Introduction

A few decades ago, flying was restricted to a small group of wealthy individuals and large public- and private-sector entities. Flying was also strictly regulated under government and international laws. However, unmanned aerial vehicles (UAVs) have changed the paradigm. Nowadays, different types of UAVs are available commercially at affordable prices, and anybody with a basic knowledge of flying can operate them. Therefore, sales of UAVs have seen a surge in the recent decade and are expected to increase in the future [1]. UAVs are used in numerous applications including search and rescue, disaster management, surveillance, package delivery, and filming [2]. UAVs are also used to measure the depth of water channels and to study coastal areas [3,4,5], where UAVs are mainly used for remote sensing and imaging, as other methods of data collection in these terrains are challenging. Moreover, UAV manufacturing is one of the fastest-growing industries in the world. The advancement of technology in other fields (e.g., smartphone manufacturing and telecommunications) has greatly benefited the UAV industry. According to a Research and Markets report [6], the UAV market is estimated to reach USD 58.4 billion by 2026, from USD 27.4 billion in 2021.

In the recent past, many incidents have been reported with malicious UAVs. There are also numerous emerging threats from malicious UAVs in the near future [7]. UAVs have inherent stealth capabilities, mainly due to their small size and ability to fly close to terrain. Therefore, malicious UAVs are difficult to detect, track, and classify. The current detection, tracking, classification, and disabling methods for UAVs are not adequate to cover a wide spectrum of threats [8,9,10]. A significant investment for finding credible solutions to countering malicious UAVs is underway by research communities and organizations all over the world [11].

Similar to UAVs, unmanned underwater vehicles (UUVs) have revolutionized sea operations. UUVs can perform many tasks that were not possible with manned underwater vehicles (MUVs) [12]. For example, UUVs can remain submerged for a very long duration of time and can operate very deep in the oceans without risking the safety of the crew, and the initial and maintenance cost of UUVs is significantly smaller compared to MUVs. Therefore, a large number of UUVs can be used at different locations in the oceans for different applications. Overall, the detection, tracking, and classification of UUVs are significantly difficult compared to MUVs [13].

In this work, we will discuss the current and possible future capabilities and features of UAVs and UUVs, the threats and challenges from malicious UAVs and UUVs at present and in the future, the limitations of the current countermeasures, and future directions for countering malicious UAVs and UUVs. The rest of the paper is organized as follows. Section 2 discusses the features and capabilities of current and expected future UAVs, while the challenges and threats from malicious UAVs are provided in Section 3. The detection, tracking, and classification of UAVs with radar-based approaches is discussed in Section 4, and in Section 5 for methods other than radar systems. Section 6 covers future directions for the detection, tracking, and classification of UAVs. Section 7 provides characteristics of UUVs, and discusses the detection, tracking, and classification of UUVs, as well as future directions for countering malicious UUVs. Section 8 concludes the paper.

## 2. Current and Future UAVs

In this section, we will discuss current UAVs and their capabilities and provide ideal features and capabilities that are expected from future UAVs.

### 2.1. Structure, Flight Characteristics, and Capabilities of Current UAVs

The absence of a pilot in an aerial vehicle helps to change the aerodynamic design and accommodate more features compared to manned aerial vehicles (e.g., high maneuverability, larger payload capacity, and flight endurance). The current design of UAVs focuses mainly on flight endurance, secured and redundant communication links, and payload capabilities [14]. However, the speed and cognitive capabilities are limited. The current UAVs can be multi-rotors or fixed-wings. The flight endurance of fixed-wing UAVs is larger compared to multi-rotor UAVs for a given amount of input energy. The longer flight endurance for fixed-wing UAVs compared to multi-rotor UAVs is mainly due to additional lift and glide from the flat fixed wings. The multi-rotor UAVs have greater maneuverability compared to fixed-wing UAVs and can hover at a single location. However, multi-rotor UAVs are inherently unstable and, from a control point of view, highly non-linear systems [15].

The remotely controlled multi-rotor and fixed-wing UAVs require a radio link [16]. The control radio link exists between the onboard UAV flight controller and the ground controller either directly, through satellites, or through other aerial vehicles in the vicinity. The data collected from a given area using onboard UAV sensors (e.g., electro-optical and infrared (EO/IR) sensors) are sent via a radio link to the ground controller. The control and data radio links have different bandwidths, and the communications are encrypted. Different frequency-hopping sequences are also used to avoid jamming. Moreover, multi-rotor and fixed-wing UAVs can fly autonomously [17]. In the autonomous mode, either the UAV follows fixed Global Positioning System (GPS)/Global Navigation Satellite System (GNSS) way-points or uses data from real-time sensors (e.g., camera, radio sensors, and stored maps) for navigation to a particular location.

The majority of the currently available UAVs, either fixed-wing or multi-rotor, use propellers for propulsion. The shape, type, and number of propellers can vary among different types of UAVs. Moreover, the UAVs can carry different payloads. The maximum payloads of popular UAVs are provided in Table 1. The payload of fixed-wing UAVs is generally larger than that of multi-rotor UAVs. The payload capacity of fixed-wing UAVs is generally increased by increasing the size of the wings. On the other hand, the payload capacity of the multi-rotor UAV can be increased by increasing the size and number of propellers [18]. The maximum speed of a multi-rotor UAV can reach 73 m/s [19], whereas the maximum speed of a fixed-wing UAV can be close to 278 m/s [20]. The maximum flight ceiling of currently available UAVs is around 25 km. (Balloons in the Google’s Loon project were designed to fly in the stratosphere, at an altitude between 18 km and 25 km [21].) Popular current UAVs, types of sensors installed, and their functionalities and features are provided in Table 1.

### 2.2. Expected Structure and Flight Characteristics of the Future UAVs

**Figure 1:**
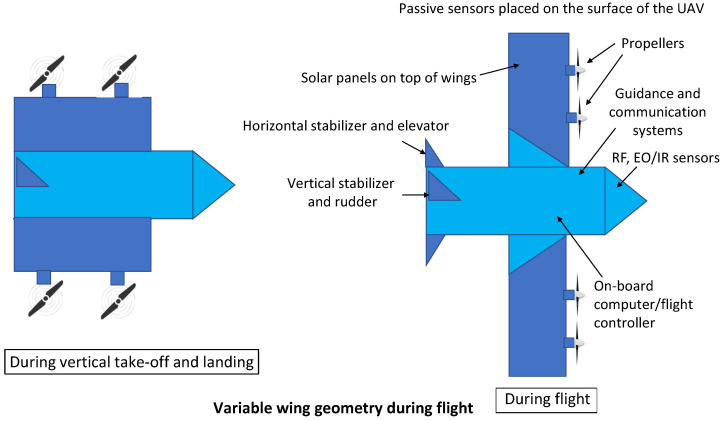
The high-level design of a futuristic UAV. Multiple sensors and sources of energy can be used. In particular, variable wing geometry can be used during different modes of flight. The variable wing geometry can help to obtain different flight speeds and to make vertical take-off and landing possible.

The structure of the UAV of the future should be simple, lightweight, modular (adaptable to different scenario requirements), and cost effective, and should allow safe landings over harsh terrain and water. New capabilities (e.g., self-healing with the help of artificial intelligence (AI) and 3D printing on-board the UAVs) can be introduced. Different sensors and sources of energy can be used. The wing geometry can be varied during different phases of the flight. The variable wing geometry can provide different speed and payload options dependent on the scenario. The variable wing geometry and propellers can also help in vertical take-off and landing, as shown in Figure 1.

The UAV should be able to carry a large payload without significantly compromising the flight duration. The long flight duration can be accomplished through the use of smart fuels, optimum fuel usage, and keeping the weight of the UAV air-frame small. Variable sources of fuel can also be used during different phases of flight. Some noteworthy fuel sources are fossil fuel, synthetic fuel, solar, hydrogen, and chargeable batteries. The UAV charging stations can be set up in high-up locations (e.g., on top of tall buildings) to save energy during take-off from the ground. The UAVs can also charge wirelessly by flying near electricity transmission lines [22]. Wireless charging using transmission lines can help to achieve a long UAV flight duration without landing.

The UAV should be able to take off and land vertically on any kind of ground surface and over water. In case of a crash, air balloons can be used to minimize the damage to the airframe. UAVs should also be able to fly in a fully autonomous mode. In the fully autonomous mode, the complete UAV flight is controlled by an AI-driven flight controller and aided by onboard sensors. In the autonomous mode, the UAV should be equipped with redundant navigation systems, which can help to mitigate external navigation reference spoofing.

### 2.3. Expected Radio and Sensing Capabilities of Future UAVs

Radio communications play an important role in UAV operations. Future UAVs are expected to support broadband radio links that implement frequency hopping to provide resilience against radio frequency (RF) jamming. There should be redundant and secured communication channels between the ground station and the UAV for important tasks. Friendly UAVs, ground stations, satellites, and other airborne platforms should be able to communicate and share sensor data over a secured link, forming a network. The UAVs should also support seamless real-time augmented reality through streaming, with UAVs in the field relaying to UAV operators, for better situational awareness.

The UAV should be equipped with different types of sensors to obtain seamless real-time information about the surrounding environment. These sensors include EO/IR sensors, lasers, and other RF sensors. Future UAVs should also be equipped with sensors to detect radio jamming, hacking, and external navigation spoofing in a given environment. In addition, countermeasures should be available against radio jamming, hacking, and external navigation spoofing. An overall comparison of current and future UAV features and capabilities is provided in Table 2.

## 3. Challenges and Threats from Malicious UAVs

In this section, we will discuss current and possible future threats posed by malicious UAVs and challenges for the detection, tracking, and classification of malicious UAVs.

### 3.1. Challenging Features of UAVs

One of the major features of UAVs is inherent stealth. The size and the infrared and acoustic signatures of UAVs are significantly smaller compared to manned aerial vehicles. Moreover, UAVs can fly at significantly low altitudes compared to manned aerial vehicles. Therefore, UAVs offer inherent stealth capabilities and are difficult to detect and track by conventional methods. The shape of small UAVs is also similar to birds. Therefore, classification of UAVs from birds is challenging [23]. Furthermore, the small energy requirement for UAVs compared to manned aerial vehicles allows long-duration non-stop flight missions.

Different types of UAVs offer different features. The features offered by fixed-wing UAVs are arguably more advantageous compared to multi-rotor UAVs. For example, the fixed-wing UAVs with delta wing configuration can carry large payloads and fly at high speeds. Fixed-wing UAVs can be programmed to fly autonomously using electronic or mechanical procedures. If mechanical procedures are used to fly the UAV autonomously, then it is immune to all kinds of electronic countermeasures (ECM). Overall, fixed-wing UAVs flying autonomously and cognitively based on terrain, and using both electronic and mechanical procedures during different phases of flight, can pose a significant challenge. On the other hand, multi-rotor UAVs are more agile compared to fixed-wing UAVs and can hover. However, multi-rotor UAVs cannot be flown only through mechanical controls.

### 3.2. Autonomous UAVs

The flight controller onboard a UAV can be configured to work autonomously [24]. The autonomous flight mode is possible with the help of onboard sensors and external navigation references (e.g., GPS, and GNSS). There are two modes of autonomous UAV flight. Either predetermined waypoints are set for UAV to follow autonomously, or AI aided by on-board sensors can be used to make real-time decisions [25].

There are many challenging features offered by autonomous UAVs compared to remote-controlled UAVs. Long-range terrain-hugging flight is possible with autonomous UAVs [26]. The terrain hugging can be performed with the help of different types of RF and EO/IR sensors. In case of terrain hugging, the detection and tracking of the aerial vehicle becomes difficult. Autonomous UAVs can also be equipped to move over land and water autonomously. The ability to maneuver over land and water can provide an alternate route in case it is not possible to continue flight in a given area. Moreover, autonomous UAVs that do not require an external navigation reference like GPS are immune to the majority of ECM. Autonomous UAVs can change shapes by integration and disintegration of parts/UAVs based on the scenario. The integration and disintegration allows UAVs to perform diverse tasks in the air and on the ground. In the future, components of autonomous UAVs, and subsequently their shape, will be capable of being modified in real time using onboard 3D printing.

### 3.3. UAV Swarms

At present, a major challenge from UAVs is when they fly in swarms. In a UAV swarm, a large number of different sizes of autonomous UAVs can be used [27,28]. The rapidly and randomly time-varying trajectories of the UAVs in the swarm make their tracking difficult. A well-coordinated UAV swarm can outperform large, expensive, and complex UAVs/manned aerial vehicles. In general, there are many possibilities where UAV swarms can be used. Some example use case scenarios are as follows:

- Each UAV in the swarm can be assigned a specific task [29], (e.g., EO/IR imaging, ECM, RF sensing). Moreover, if the payload is large, then it can be divided into modules and carried separately by individual UAVs in the swarm and can be combined during the flight when required.
- In a UAV swarm, each UAV can either fly autonomously [30] following a pre-planned trajectory or cognitively adopt a trajectory [31] based on the real-time scenario using on-board sensors. AI algorithms can be used by each UAV in the swarm to coordinate with each other and/or the central controller. The central controller can be on the ground, a UAV in the swarm, or a manned aerial vehicle.
- UAV swarms can adopt different shapes in the air [32] and can be equipped with the ability to integrate and disintegrate in the air when required.
- UAV swarms can be used as airborne assets that can provide better situational awareness [33].
- A swarm of UAVs in the air can also be used to create an antenna array [34]. Each UAV can carry an antenna element. The antenna array and subsequently the radiation pattern can be reconfigured by changing the position of the UAVs.
- Miniaturized UAVs that have dimensions of a few inches can also be used in swarms [35]. The miniaturized UAVs in a swarm can work similarly to honey bees. There is a small effect of the environment on the flight of miniaturized UAVs (e.g., wind gusts). Miniaturized UAVs can also integrate to form large devices in the air in real time (e.g., to display patterns or to form a mobile phased array antenna in the air).

### 3.4. Electronic Countermeasures

Offensive and defensive electronic countermeasures (ECM) can pose a significant challenge to the detection, tracking, and classification of malicious UAVs. The UAVs can be equipped with various offensive and defensive ECM. Popular offensive ECM are jamming, spoofing, hacking, and high-energy radiation burst. The defensive ECM helps to shield against offensive ECM. Some popular defensive ECM are as follows:

- The RF link between the UAV and the remote controller is vulnerable to jamming [36] and hacking [37]. The jamming can be avoided by spread-spectrum techniques and high frequency-hopping rates. The hacking attempts can be thwarted by using multi-layered authentication and encryption. The threat of jamming and hacking can also be reduced by using redundant RF links.
- RF cognitive techniques can be used to analyze the energy distribution and hopping patterns of the RF jammer [38]. The analysis can help to adjust the RF parameters onboard the UAV accordingly to avoid jamming.
- GPS spoofing can be eliminated by identifying the spoofed GPS signals [39]. The comparison of signals from multiple navigation references (both internal and external) can help to identify the spoofed GPS signal.
- To save UAV onboard electronic equipment from high-energy electromagnetic (EM) radiation burst, metallic shielding can be used. Metallic and lead shielding can offer protection for the onboard electronic equipment against the EM radiation burst.

### 3.5. Types of Threats from Malicious UAVs

Conventional threats were perceived mainly from ground-based targets, and security measures were designed accordingly. However, UAVs have added another dimension to the conventional threats spectrum. Many threat scenarios can arise using UAVs. The major threats that involve malicious UAVs include [40,41,42,43]:

- Hazardous payloads that can be carried by mischievous UAVs for long distances.
- Surveillance and intelligence gathering by malicious UAVs (e.g., UAVs can be used to gather information near sensitive locations like police stations, borders, etc.).
- Illegal activities using UAVs. Numerous activities can be carried out by using mischievous UAVs (eavesdropping, stealing personal data, unauthorized imaging, identity theft, and starting a fire, etc.).
- Threats to governmental authorities, vehicles, and infrastructure. In particular, malicious UAVs can present a significant threat to sensitive infrastructure (e.g., nuclear power plants and chemical plants).
- Threats to crowded areas.
- Threats to the civil aviation industry. In the recent past, there were many reported incidents of mischievous UAVs interfering with the civilian flight operations [44].
- UAV swarms. At present, the threat of UAV swarms is difficult to counter.
- Unauthorized control of UAVs while flying. Hacking UAVs and flying them for malicious purposes.

## 4. Radar Systems for UAV Detection, Tracking, and Classification

Radar systems are mainly used for the detection, tracking, and classification of UAVs. In this section, we describe the detection, tracking, and classification of UAVs using radar systems. The limitations of the radar systems for the detection, tracking, and classification of UAVs are also discussed.

### 4.1. Conventional and Advanced Radar Systems

**Figure 2:**
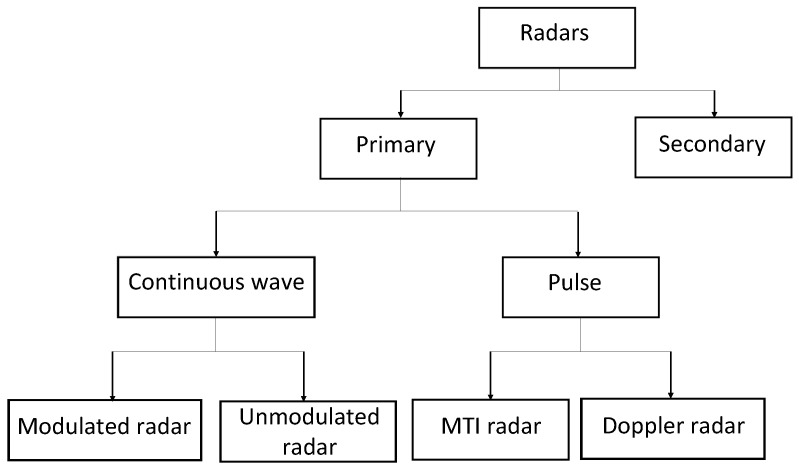
Different types of radars for detection, identification, and tracking of aerial vehicles. Primary radars are used for detection of aerial objects, whereas secondary radars are used for identification. Primary radars are sub-categorized into continuous wave and pulse radar.

The performance of a radar system in a given terrain and for a given scenario (e.g., the type and number of aerial vehicles) depends on many factors including center frequency, bandwidth, modulation, sounding signal, pulse repetition frequency, receiver sensitivity, antenna beam characteristics, and clutter rejection ability [45]. The two main types of radar systems are pulse and continuous wave. Pulse and continuous wave radars are further sub-categorized as shown in Figure 2. Radar systems are generally configured/calibrated for conventional manned aerial vehicles, and modifications are required to detect, track, and classify small UAVs. Conventional radar systems use popular frequency bands including very high frequency (VHF), ultra high frequency (UHF), L, S, C, X, Ku, K, Ka, and millimeter wave.

In addition to conventional radar systems, there are advanced radar systems for detection, tracking, and classification of UAVs. Among the advanced radar systems, active electronically scanned array (AESA) radars are popular for the detection of small UAVs [46]. Advanced radar systems include passive, hybrid, multiple-input and multiple-output (MIMO), and cognitive radars. Passive radar systems can use different indirect illuminations (e.g., TV and FM radio broadcasting) for detection and tracking of UAVs [47,48,49,50]. Hybrid radar systems can also be used for precise positioning, detection, and tracking of aerial vehicles. In [51], a hybrid radar system using frequency-modulated continuous waves (FMCW) and interferometry is used for precise positioning. Similarly, in [52], a hybrid radar system consisting of active and passive radars is described. The hybrid active and passive radars in [52] provide better detection performance compared to single active/passive radar systems.

**Figure 3:**
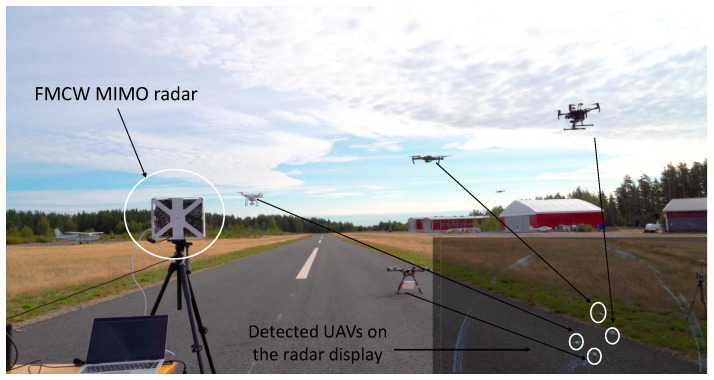
Detection and tracking of multiple UAVs with the help of 60 GHz FMCW frequency-division multiplexing MIMO radar [57].

MIMO and cognitive radar systems can also be used for the detection and tracking of UAVs [53,54,55,56]. MIMO radars can provide better detection and tracking of small UAVs due to spatial diversity, which helps to improve ground clutter rejection. A 60 GHz FMCW MIMO radar [57] is shown in Figure 3. The FMCW MIMO radar in [57] is capable of detecting hovering and moving aerial vehicles simultaneously at tens of meters of range; however, UAV detection is not the main application of this radar, and it is included here rather for demonstration purpose. The main advantages of MIMO radar are high frame rate and ability to collect micro-Doppler data from multiple aerial vehicles simultaneously. Cognitive radars are also helpful in the detection and tracking of modern aerial threats. The parameters of the cognitive radar can be selected to be adaptively dependent on the aerial vehicle and the scenario. Adaptive parameter selection greatly helps in the detection, tracking, and classification of UAVs [58]. In addition, light detection and ranging (lidar) can also be used for the detection and tracking of UAVs [59]. Lidars can separate the background scene from a desired UAV easily compared to EO/IR imaging. The performance of UAV detection using lidar is better compared to other detection techniques in urban and cluttered environments. However, scanning using lidar takes a significant amount of time.

### 4.2. Detection of UAVs Using Radar Systems

The detection of an aerial vehicle by a radar system depends on the radar cross-section (RCS) of the aerial vehicle. The RCS of an aerial vehicle depends mainly on the frequency, polarization, and aspect angle. The RCS also depends on the physical characteristics of the aerial vehicle. The physical characteristics include the geometry and material of the aerial vehicle [60]. UAVs have significantly small RCS mainly due to their small size and largely non-metallic bodies. Small-RCS and low- and slow-flying UAVs are difficult for conventional radar systems to detect at long ranges without system calibrations [61]. The maximum range $R_{\max}$ obtained using a radar after detection is given by the following:

(1) $$R_{\max}=\sqrt[{4}]{\frac{P_{T}G^{2}\lambda^{2}\sigma }{P_{R,\min}{\left(4\pi \right)}^{3}L}},$$

where $P_{T}$ and $P_{R,\min}$ are the transmit power and the minimum received power, respectively; *G* is the antenna gain; λ is the wavelength; σ is the RCS; and *L* represents the internal losses of the radar.

Detection using radar systems can be broadly classified as active or passive. Active radar systems are mainly used. Active radars have longer detection ranges and better tracking and classification compared to passive radars. Long-range detection is carried out using active search radars. The range and angular resolution of search radars are generally low compared to tracking/guidance radars. High-range and high-angular-resolution (but limited-range) active search radars using AESA or passive electronically scanned array (PESA) [62] can also be used. In comparison to active radars, passive radars/receivers use radio emissions from other radars or communications sources in the vicinity to detect the presence of an aerial vehicle. Passive radars are also popular for the detection of stealth aerial vehicles [63].

There are different radar systems for the detection of UAVs available at present [64,65]. FMCW, pulse-Doppler, ultra-wide-band, and phase interferometry radars are popular for the detection of UAVs [66,67,68,69]. AI techniques are also used for the detection of multiple UAVs simultaneously [70]. Similar to radar, lidar is helpful for the detection of UAVs [59].

### 4.3. Tracking of UAVs Using Radar Systems

Tracking of aerial vehicles can be performed either by a search radar or by a separate tracking radar [71]. A dedicated tracking radar is generally used to obtain continuous information of the aerial vehicle using narrow illumination beams [71]. Tracking radars have higher range and angular resolution beams compared to search radars. The tracking equation of radar is as follows:

(2) $$S/N=\frac{P_{t}G^{2}\lambda^{2}\sigma }{{\left(4\pi \right)}^{3}R^{4}kT_{s}B_{n}L},$$

where S/N is the signal-to-noise ratio, *R* is the slant range between the radar and the aerial vehicle, *k* is the Boltzmann constant, $T_{s}$ is the total system temperature, and $B_{n}$ is the RX noise bandwidth. During tracking, the position coordinates, in 2D or 3D, and motion characteristics (velocity and trajectory) of the aerial vehicle are stored. The position coordinates includes range, azimuth, and elevation angles of the aerial vehicle from the radar reference.

Numerous techniques for UAV tracking are available in the literature [72,73]. The tracking of UAVs is challenging compared to manned aerial vehicles at long distances, mainly due to the small size of UAVs [74]. Trajectory tracking of highly maneuverable UAVs is discussed in [75], where a filtration algorithm is used for UAV trajectory tracking. The tracking using filtration algorithm in [75] provides better tracking of highly maneuverable UAVs in noisy environments compared to other techniques for tracking. Moreover, tracking of multiple aerial vehicles is complicated compared to single aerial vehicle tracking [76]. For example, the tracking becomes difficult for a large swarm of UAVs [77].

Based on the tracking information, future trajectory estimates are made using estimation algorithms [78]. The trajectory estimation helps in: (1) estimation of the point of the origin of the desired aerial vehicle; (2) estimation of the terminal point; (3) guidance towards an aerial vehicle; (4) target classification based on the trajectory features; and (5) predicting the intent of the aerial vehicle. AI algorithms are mainly used to forecast the trajectory of UAVs [79,80]. In addition to AI algorithms for trajectory estimation, other algorithms are available in the literature. In [81], dynamic Bayesian filters are used for tracking and estimation of UAV trajectory.

### 4.4. Classification of UAVs Using Radar Systems

Classification of aerial vehicles using conventional radar systems was not very refined. For example, the RCS and velocity of aerial vehicles were used for classification of different types of aerial vehicles. However, the exact classification of the type of aerial vehicle was not always made. Nowadays, aerial vehicles have grown in complexity and types, and new classification methods are available using radar systems [82]. In particular, the classification of UAVs compared to birds is challenging due to similar features [83]. Many techniques are available in the literature for the differentiation of small UAVs from birds [84,85].

**Figure 4:**
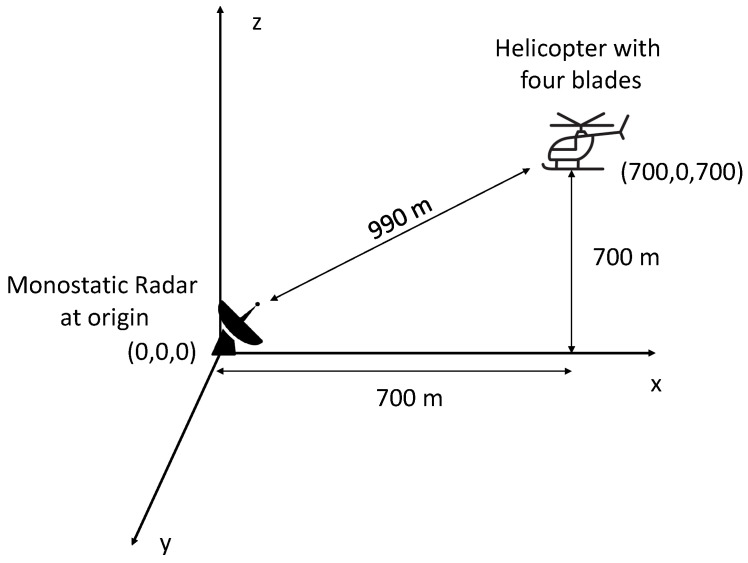
A micro-Doppler simulation scenario using a monostatic radar and four-blade helicopter [90]. The monostatic radar is immovable and is placed at the origin. The initial helicopter coordinates are (700,0,700). The blades of the helicopter rotate at four revolutions per second, and the velocity of the helicopter is 80 m/s.

**Figure 5:**
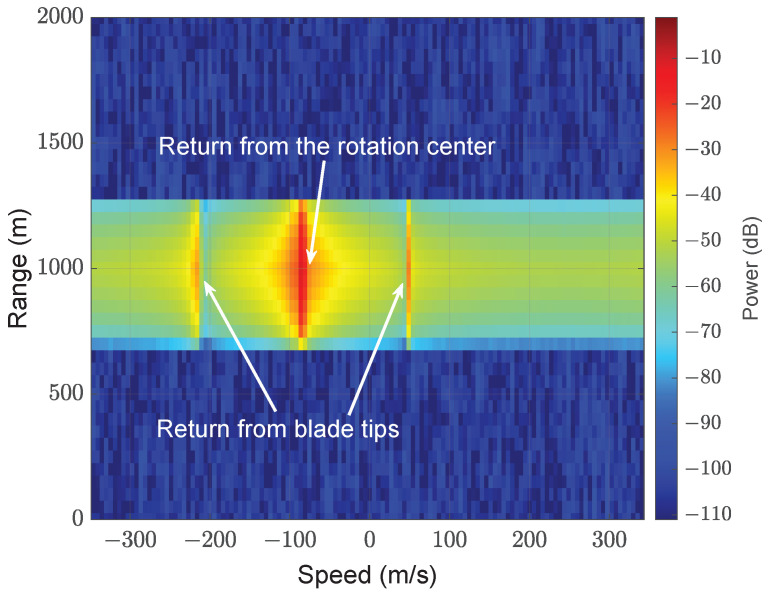
The range-velocity plot of the setup is shown in Figure 4 (reprinted from [90]). The major power concentration is centered at (66.6 m/s, 975 m) due to the central rotation part of the helicopter. The other two minor concentrations are due to the radar returns from the tips of the blades.

**Figure 6:**
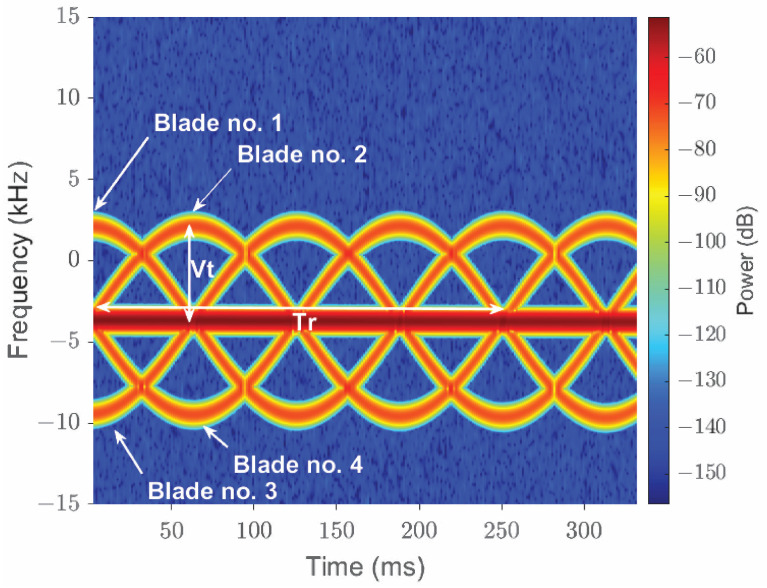
Micro-Doppler modulation due to (1) rotating blades and (2) a constant Doppler shift from the central rotating part of the helicopter (reprinted from [90]). The sinusoidal behavior is due to the rotation of the blades.

The features of different types of UAVs also differ significantly [86]. These features are used for classification. For example, micro-Doppler radar signatures are used for enhanced detection of multi-rotor UAVs [87] and to differentiate small UAVs from birds [23]. In [88], experimental micro-Doppler signatures are studied to classify payload and intent. A micro-Doppler setup scenario for a four-blade helicopter and monostatic radar is shown in Figure 4. Range-velocity, micro-Doppler modulation plots are shown in Figure 5 and Figure 6, respectively. From Figure 5 and Figure 6, it can be observed that there are distinct contributions from the rotating blades and rotating central body (where the blades are attached). The micro-Doppler signatures in Figure 5 and Figure 6 help to classify the aerial vehicle.

The classification of UAVs using radar systems is greatly aided by AI algorithms [64]. In [89], laser mesh in the air is simulated to obtain the parameters of aerial vehicles. Different classification models are generated based on the AI classifiers, training data, and aerial vehicle classes in [89], given as follows:

(3) $$\begin{array}{c} M_{1}=f^{\left(\mathrm{NB}\right)}\left(M^{\left(\mathrm{Tr}\right)},C^{\left(\mathrm{Tr}\right)}\right),\;M_{2}=f^{\left(\mathrm{LDA}\right)}\left(M^{\left(\mathrm{Tr}\right)},C^{\left(\mathrm{Tr}\right)}\right),\;M_{3}=f^{\left(\mathrm{KNN}\right)}\left(M^{\left(\mathrm{Tr}\right)},C^{\left(\mathrm{Tr}\right)}\right), \end{array}$$

where $M_{i}$ for i=1,2,3 are the classification models based on the naive Bayes, linear discriminant analysis, and K-nearest neighbor classifiers; $f^{\left(\mathrm{NB}\right)}$, $f^{\left(\mathrm{LDA}\right)}$, and $f^{\left(\mathrm{KNN}\right)}$ are the classifier functions for naive Bayes, linear discriminant analysis, and K-nearest neighbor, respectively; $M^{\left(\mathrm{Tr}\right)}$ is the training matrix containing training data; and $C^{\left(\mathrm{Tr}\right)}$ is the array containing the aerial vehicle classes in [89]. The estimation of a particular class using the predict function is given as follows:

(4) $$C^{\left(\mathrm{est},M_{i}\right)}=\mathrm{predict}\left(M_{i},M^{\left(\mathrm{eval}\right)}\right),$$

where $M^{\left(\mathrm{eval}\right)}$ is the matrix containing the feature data of the aerial vehicle for evaluation.

### 4.5. Limitations of Radar Systems

There are various limitations of the radar systems for detecting and tracking modern aerial threats [91], mainly from UAVs. Similarly, offensive ECMs applied by UAVs provide a major challenge to any modern radar system. Limitations of radar systems are summarized as follows:

- Radar systems are generally inefficient in terms of the energy transmitted and received. A large amount of transmit energy is required for the detection of small UAVs.
- Active radar systems have the risk of detection due to energy emission.
- There are limitations of the radar systems for detecting, tracking, and classifying small, low-flying UAVs in cluttered scenarios [92].
- The detection of small RCS aerial vehicles (e.g., UAVs) requires careful calibration of the radar system and constant adjustment of the detection threshold. The pfa for small-RCS aerial vehicles in cluttered environments generally increases [93].
- A large swarm of UAVs can overwhelm the majority of radar systems. The individual UAVs in a swarm are difficult to track by radar systems. Range and Doppler ambiguities are expected to increase for a given radar system detecting a large swarm of UAVs.
- Offensive ECMs used by UAVs can jam and spoof radar systems.

## 5. Methods Other Than Radar Systems for UAV Detection, Tracking, and Classification

Due to limitations of the radar systems, as discussed in Section 4.5, there are numerous methods proposed in the literature for countering malicious UAVs. In this section, current methods of detection, tracking, and classification of UAVs other than radar systems are discussed. Current methods of disabling UAVs are also provided.

### 5.1. EO/IR

The popularity of EO/IR methods has increased in the recent decade [94]. The EO/IR imaging technologies can operate at different frequency bands. The selection of the frequency bands is dependent on the type of aerial vehicle and operating environment. EO/IR sensors can provide excellent detection, tracking, and classification of small UAVs in day/night conditions [95,96]. However, the performance of EO/IR sensors is dependent on the complexity of the background of the aerial vehicle and the thermal image saturation. Haze, fog, rain, and snow can also affect the performance of EO/IR imaging sensors. The EO/IR imaging technique has limitations in detecting small UAVs in highly cluttered environments. Moreover, the range of EO/IR imaging is limited by the horizon. The range of EO/IR imaging can be increased with the height of the sensors above the ground.

### 5.2. RF Analysis

**Figure 7:**
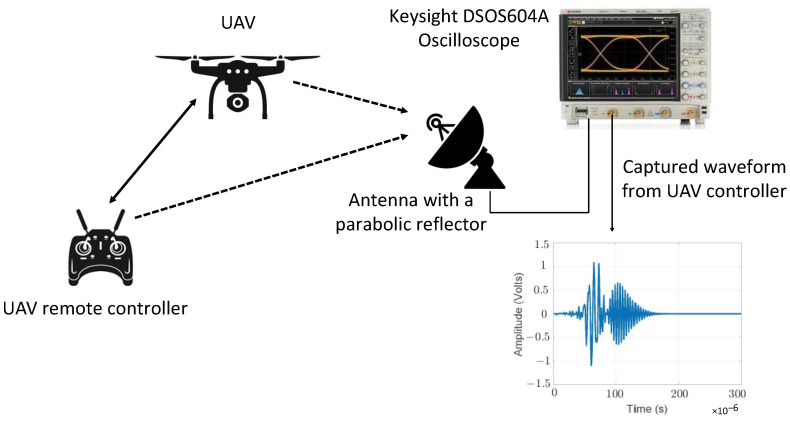
Setup for detection of a UAV using RF analysis. The RF link is between the ground controller and flight controller. Based on the energy detection of the RF link, a UAV can be detected and subsequently classified (reproduced from [97]).

The RF signature of signals between the UAV ground controller and the UAV onboard receiver is different for different types of controllers [97,98]. The particular signature or fingerprint of the RF link between the UAV ground controller and the onboard UAV receiver can be sensed passively to detect the presence of a UAV. The RF fingerprint of the communication link between the UAV ground controller and the UAV receiver is unique compared to other wireless communication devices. RF fingerprinting can also be used to determine the type of the UAV [99]. In [97], the RF link between the UAV and ground controller is analyzed, and machine learning methods are used for the classification of a particular type of ground controller. The RF analysis setup in [97] is shown in Figure 7. RF fingerprinting for the detection and classification of UAVs is only effective if there is an active RF link between the UAV ground controller and the UAV receiver. Autonomous UAVs that have no external RF link can fly in radio silence and cannot be detected by RF fingerprinting.

### 5.3. Acoustic Analysis

Different types of UAVs have different acoustic signatures. The acoustic signals from the propellers, engine, and motors are used to detect the presence of a UAV [100,101]. Microphones are used to collect the sound/noise from the flying UAV. The acoustic signals and AI algorithms can also be used to determine the type of a UAV [102]. Similar to RF fingerprinting, acoustic sensing works in a passive mode for the detection of UAVs. The major limitations of the acoustic approach are limited range and performance dependence on ambient noise.

### 5.4. Sensor Fusion

A combination of different sensors discussed above can be used for the detection, tracking, and classification of UAVs [103]. Sensor fusion can help to provide layered security around a given parameter. A network of different sensors placed at different locations can provide better spatial coverage compared to a single-location placement of a large sensor. The sensors can be placed on the ground or mounted on an airborne platform (e.g., a UAV or satellite). Airborne sensors have better visibility and coverage compared to ground-based sensors. Airborne sensors can also move to optimum locations based on the scenario.

### 5.5. Current Methods for Disabling UAVs

The current methods for disabling UAVs are divided into kinetic and non-kinetic methods [104]. The kinetic methods mainly use nets, prey birds, and guided or unguided projectiles. There are limitations of using nets and prey birds for countering UAVs. Nets have a limited range and require a precise tracking system to determine the exact coordinates of the desired UAV in space. The use of a net may not be effective against several spatially spaced UAVs. Countering UAVs with large prey birds is also challenging. The initial cost, training, and maintenance cost of prey birds is large. Prey birds are not effective in certain weather and light conditions. The prey birds also have limited efficacy against UAV swarms.

The non-kinetic methods include (1) GPS spoofing [39], (2) hacking RF control communications between the ground controller and the UAV [105], (3) high-energy EM radiation burst [106], and (4) high-energy laser [107]. There are limitations of non-kinetic methods also in disabling UAVs. GPS spoofing and hacking RF communications are not effective against autonomous UAVs that have no external RF link and require no external navigation reference. The high-energy EM radiation method also has limitations. High-energy EM radiation may be ineffective against electronic equipment that is shielded. In addition, the range of high-energy EM radiations is limited, and the cost is high. High-energy lasers may not be effective against a wide spectrum of UAV threats. A high-energy laser is less effective against highly mobile UAVs equipped with heat sensors. High-energy laser beams may also be ineffective against UAV swarms. Moreover, the initial cost and energy required for the laser beams are high.

## 6. Future Research Directions for UAV utilization and their Detection, Tracking, and Classification

The current countermeasures are not always effective against a broad spectrum of threats from UAVs. There are various research efforts to find novel methods to counter malicious UAVs. In this section, future directions for the potential uses of UAVs and their detection, tracking, and classification are discussed. A comparison of current and possible future countermeasures against malicious UAVs is provided in Table 3.

### 6.1. Potential Uses of UAVs

Smart UAVs are expected to revolutionize the evolving UAV technology and potential future applications. A major application of smart UAVs is in the field of civil infrastructure. The civil infrastructure market is expected to cover more than USD 45 billion of the UAV market share [108]. In particular, laser power beaming is envisaged to provide supplemental energy to smart UAVs when the solar energy is not adequate. Laser power beaming can allow smart UAVs to fly for weeks and months without landing [108]. In [109], use of intelligent UAVs to enhance the economic growth of a specific world region is discussed. The major sectors of the economy where intelligent UAVs can be used include precision agriculture, environmental pollution monitoring, geophysical processes monitoring, exploration of minerals, and monitoring of traffic and cattle/wild animals. The possibilities of UAVs used as remote sensing nodes for solving urban area issues are discussed in [110]. The use of UAVs as remote sensing nodes can provide solutions to different urban area issues, such as collection of data for urban planning, disaster management, and infrastructure monitoring. UAVs are also envisioned as an integral part of future smart cities [111]. UAVs in future smart cities can be used for traffic management, environmental and pollution monitoring, civil security management, and goods delivery.

### 6.2. Modern Radar Systems

There are new radar systems available in the literature for the detection of UAVs, such as MIMO radars with narrow beams and high angular resolution that are effective against small UAVs [53]. MIMO radar systems can be mounted on airborne platforms that can provide better spatial diversity and situational awareness. Moreover, reconfigurable antennas can be used for MIMO radars for better adaptability to a given scenario. Cognitive radars can also be effective against UAV threats [58]. Cognitive radars can select the transmit signal characteristics, illumination of the aerial vehicle, and receiver characteristics based on feedback from the environment and aerial vehicle. Cognitive radar systems are expected to gain significant attention in the future for detection, tracking, and classification of UAVs. Future cognitive radar systems are expected to have reconfigurable antennas and AI-driven cognitive engines.

**Figure 8:**
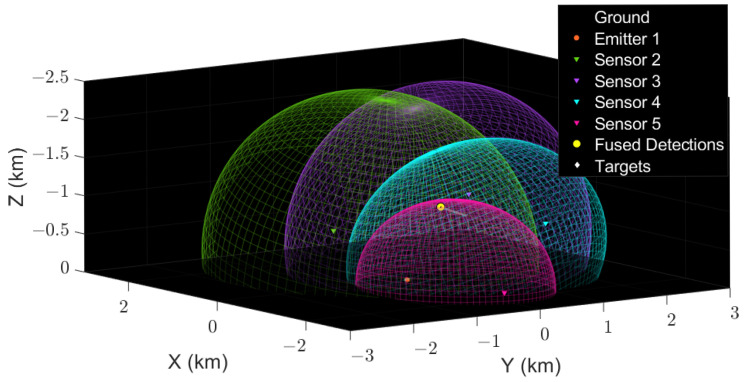
Bi-static sensors placed at different locations are shown as inverted triangles for aerial vehicle detection (reprinted from [117]). Four ellipsoids indicate the coverage of the respective bi-static sensors. A single emitter is shown as a purple circle at the origin. The aerial vehicle is shown as a white diamond wrapped around by a yellow circle indicating fused detections. The fused detections due to the overlapping of the ellipsoids are close to the actual position of the aerial vehicle. The trajectory is shown as a grey line.

Passive radars have also gained popularity for the detection of small UAVs and stealth aerial vehicles [112,113,114,115]. Different sources of illumination (e.g., TV, FM, and telecommunications signals) can be used. Passive radars in bi-static and multi-static configurations can also be used for early warning against UAVs [48,116]. A bi-static radar system with one emitter and four receivers/sensors is shown in Figure 8 (from [117]). In Figure 8, four ellipsoids indicate the coverage of the bi-static sensors. The fuse detection lies close to the intersection region of the ellipsoids and close to the actual position of the aerial vehicle. The estimated range $R_{b}$ using bi-static radar is given as follows [118]:

(5) $$R_{b}=\sum_{j=-2}^{2}R_{c}\left(d+j\right).z{\left(d+j\right)}^{2},$$

where $R_{c}\left(x\right)$ is the range at the range bin center; *x*, z(x) is the range cell voltage at cell *x*; and *d* is the range cell where detection is declared. Multiple antennas and polarization can be used for future passive radars for reception of scattered signals from UAVs. Moreover, beam steering can be used for passive radars that can help to receive weak aerial vehicle signals from a given direction.

Other modern radars include interferometry radars [69,119] for detection and tracking of UAVs. A combination of different radar techniques can also be used for the detection of modern aerial threats [51,52,85]. Micro-Doppler radars are popular for UAV classification [88]. The micro-Doppler radar returns are processed using various AI algorithms for the classification of UAVs [120]. A major limitation of micro-Doppler radars is their short range, high sampling rate requirement, and long post-processing time [82]. These limitations need to be overcome in future UAV classification applications.

### 6.3. AI Techniques

AI algorithms can help in the detection, tracking, and classification of UAVs using popular approaches, such as radar [121], EO/IR [122,123], acoustics [70], and RF analysis [98]. The detection and classification of UAVs using AI algorithms require a large database of training data. The training database can consist of size, shape, payload, flight characteristics, micro-Doppler, RF, infrared, and acoustic signatures of different types of UAVs. Major limitations of the AI techniques for detection, tracking, and classification of UAVs are the large training data and processing time requirements [124]. Future AI techniques for detection, tracking, and classification of UAVs are required to overcome these limitations by using smart training data and efficient AI algorithms.

Different types of UAVs follow specific trajectories for different flight trips. The trajectory of a UAV can be used to forecast the future track and intent of the UAV. A database of the trajectories of different types of UAVs for different flight operations can be created. The database and AI algorithms can be used to forecast the trajectory and intent of a detected UAV in real time. Moreover, the UAV trajectory estimates can be improved by combining trajectory estimates collected from multiple sensors at different spatial locations.

### 6.4. Laser Beams

**Figure 9:**
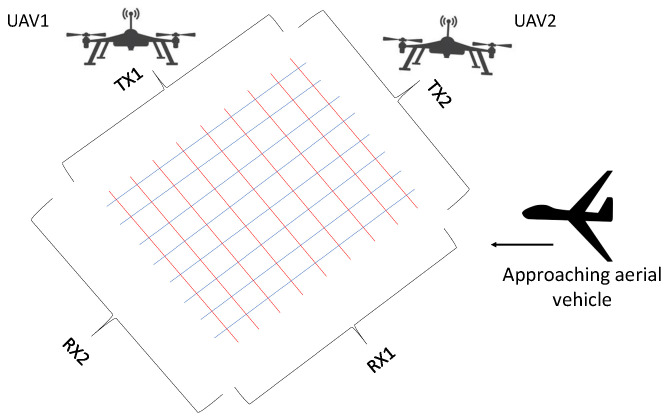
A mesh of laser beams was created using two airborne UAVs (reprinted from [89]). Each airborne UAV produces a uniformly spaced array of laser beams. A mesh is formed by the appropriate positioning of the airborne UAVs. The laser mesh can be used to detect, track, and classify different types of targets [89].

The concept of using laser beams to detect, track, and classify different types of aerial vehicles is new. It was introduced in [89]. A mesh of laser beams can be created using at least two of the airborne platforms described in [89] and shown in Figure 9. The laser beams extend from the airborne platform to the ground. Any aerial vehicle that blocks the path of the laser beams will be detected and subsequently localized and classified. A major benefit of the laser mesh is that an aerial vehicle will be detected independent of the size, shape, and material. Moreover, the tracking and classification will be significantly simplified compared to radar systems. However, a major limitation of the laser beams setup is the large initial deployment cost. A possible solution to large initial cost is to use airborne infrastructure (e.g., satellite constellations) for dual purposes.

### 6.5. Space and Airborne Assets

Space and airborne assets (e.g., satellites, high-altitude tethered UAVs) can be used for long-range surveillance and can provide large area coverage and early warning against low-flying aerial vehicles. Different types of sensors can be used on space and airborne assets. For example, cameras and RF sensors can be used on tethered UAVs placed at different locations of important areas (e.g., airports) for additional visibility and better situational awareness. A major bottleneck for the operations of the airborne sensors is the wireless link between the airborne sensor and other communicating nodes. The wireless link between the airborne sensor and other communicating nodes is vulnerable to ECM (e.g., jamming and hacking). The operational cost of sensors onboard airborne platforms is also high compared to ground platforms. In the future, large satellite constellations could be used for remote UAV localization, in addition to other tasks.

### 6.6. Distributed Sensors

**Figure 10:**
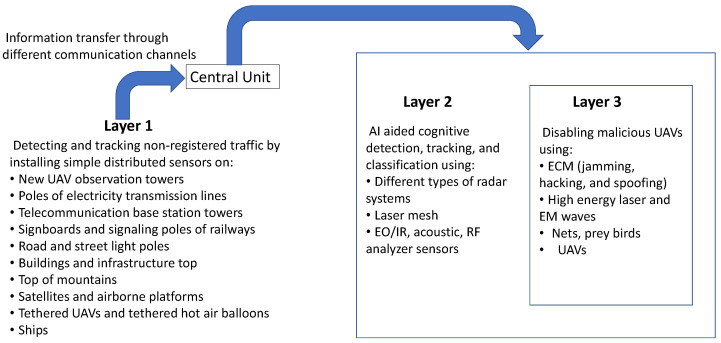
In addition to conventional layers for countering UAVs (layers 2 and 3), we have introduced layer 1 sensors. The first layer sensors are placed at different locations. The first layer can help in the early detection, tracking, and classification of intruding UAVs.

A layer of distributed sensors can be used for the early detection, tracking, and classification of UAVs. Small, simple, and off-the-shelf sensors can be used over a large area for the detection, tracking, and classification of UAVs. Small and off-the-shelf sensors distributed over a large area can compensate for large, complex, and expensive sensors installed at single or limited locations. For example, microphones and/or low-cost cameras can be installed on the rooftops of tall buildings, cellular towers, electricity transmission poles, and signboards for early detection, tracking, and classification of passing UAVs. A possible scenario using layers of distributed sensors is shown in Figure 10. In Figure 10, the sensors are used at three layers. The output of the first-layer sensors is fed to a central network that can provide early warning to the subsequent layers of sensors. A major limitation of using a distributed sensors network for UAV detection and tracking is the relay of information from distributed points to a central location. A possible solution to this limitation is to use already available telecommunication networks for information relaying.

### 6.7. Countering UAV Swarms

Radars can be more effective in the detection of UAV swarms at a large distance compared to other methods. However, radar systems have difficulty in tracking a large swarm of spatially spaced aerial vehicles simultaneously. In addition to current techniques available in the literature for detecting and tracking UAV swarms, a possible solution is to use area-based tracking. Multiple steerable antenna beams can be used for area-based tracking. Each beam can track a 3D area occupied by a cluster of UAVs instead of tracking individual UAVs. Each 3D area will contain a different number of UAVs at different scanning instances. Area-based tracking can better assist in tracking individual UAVs in a swarm.

### 6.8. Regulating UAV Traffic

In addition to the techniques available in the literature for UAV traffic management [125], in the future, aerial highways for UAVs can be envisioned. The UAVs can only pass through the aerial highways and will be tagged and registered. The tagged and registered UAVs will be considered legitimate traffic. An IP address is assigned dynamically to the UAV, and its international identification address will be registered before entering the aerial highway. Any UAV that does not follow the route can be fined and taken away from the air. A new base-station-like structure and available telecommunication base stations can be used as checkpoints. As the UAV moves across the aerial highways, its correspondence will be handed over to multiple checkpoints. In addition, every UAV should have a mandatory transponder installed for identification during the flight.

### 6.9. Terrain-Specific Countermeasures

The countermeasures effective in one terrain may not be effective in another. For example, in an urban environment, the acoustic detection method may not work efficiently. Similarly, short-range radar systems using the higher end of the spectral band may have difficulty detecting aerial vehicles in a mountainous area. Therefore, the countermeasures for UAVs can be adopted based on the specific terrain. In addition, long-range detection, tracking, and classification of UAVs in different terrains can be performed using assets in space. An example is a satellite multi-static radar system. The transmitter of the satellite multi-static radar can be located on the satellite, and the receivers can be placed at distributed locations on the ground to collect the reflected signals from UAVs. Terrain-specific countermeasures may work better compared to multi-terrain countermeasures; however, a specific countermeasure in a given terrain may not be fully effective in another terrain.

## 7. Unmanned Underwater Vehicles

In this section, the current and expected future capabilities of UUVs are described. The challenges and threats from intruding UUVs, and the detection, tracking, and classification of UUVs using active and passive sonar are also covered in this section.

### 7.1. Capabilities of Current and Future Unmanned Underwater Vehicles

The long duration and low-cost deepwater missions possible using UUVs have changed the paradigm of sea operations in favor of UUVs [126]. UUVs are similar to MUVs in shape and capabilities. However, the absence of humans allows a smaller size; higher maneuverability; low initial, operational, and maintenance costs; larger payload; higher maneuverability and speed; deepwater operations; longer duration; and quieter operation compared to MUVs. The small cost of UUVs compared to MUVs allows using multiple UUVs at different locations. The UUVs at different locations can form a network for sharing information. For example, the UUVs can be used in swarms [127] that can work as a distributed sensor network covering vast areas and various depths undersea. UUVs in a swarm can be used for different applications, such as underwater communications, surveillance, decoys, and escorts for underwater and surface vehicles. Moreover, simple sails can be used to convert UUVs into overwater unmanned vehicles. In Table 4, popular autonomous UUVs, types of sensors installed, dimensions, maximum endurance, and operating depths are provided.

UUVs can either be controlled remotely or work autonomously using AI. However, the communication link for remote control of UUVs has many challenges underwater. Radio waves cannot travel deep into the water, and acoustic communications have small bandwidth and large delays. Therefore, the majority of UUV operations are autonomous [128]. Autonomous UUVs face difficulties in transferring real-time information remotely while submerged. In addition, in the case of a robotic failure (e.g., due to a sudden change in the sea conditions), the UUV is lost in the majority of cases.

Another limitation of UUVs is the choice of energy source. Currently, battery-powered UUVs are used that have limited time duration underwater. Glider UUVs, on the other hand, requires a small amount of energy and can be used for long-duration operations [129]. However, glider UUVs are required to follow a specific up and down motion. Navigation reference for positioning is also challenging for UUVs underwater. Long baseline [130] and ultra-short baseline [131] are commonly used for positioning reference underwater. However, a UUV needs to be in the coverage area of the long and ultra-short baseline position systems, and the accuracy is dependent on the underwater position of the UUV.

Future UUVs are expected to have the following capabilities: (1) multiple communication links operating at different spectral bands to transfer real-time underwater data; (2) energy-efficient long-duration operations; (3) multiple sensors installed and controlled cognitively; (4) high positioning accuracy; (5) ability to communicate with nearby UUVs, forming a network; and (6) ability to operate underwater and overwater.

### 7.2. Challenges and Threats from Malicious UUVs

Compared to manned underwater vehicles, UUVs are difficult to detect, track, and classify undersea. The challenges in the detection, tracking, and classification of UUVs are mainly due to their small size (similar to common marine animals), deepwater operations, small noise generation while moving underwater, and the ability to remain submerged for a long period of time. Furthermore, UUVs coated with sound-absorbing material, able to move both over and under the sea, and equipped with autonomous cognitive abilities, present additional challenges to detection, tracking, and classification. The mischievous UUVs that work autonomously present major threats, and the threats can be categorized as follows:

- Threat of unauthorized surveillance using malicious UUVs [132]. For example, UUVs can move close to coastal areas comparatively easily compared to manned underwater vehicles. While near the coastal areas, the UUVs can eavesdrop on the surroundings. Similarly, mischievous UUVs can be used to gather surveillance information while at sea by attaching to a large ship as a parasite.
- Threat of unauthorized smuggling using malicious UUVs [133]. Malicious UUVs can be used to carry contraband items undersea without detection.
- Threat of spoofing and jamming using mischievous UUVs. Malicious UUVs can be used to produce spoofed GPS signals that can divert ships. Malicious UUVs can also be used to hack/deny information between ships or between ship and air/ground transceivers.
- Threat from a swarm of malicious UUVs. UUVs can operate autonomously in a swarm underwater [134,135]). A swarm of malicious UUVs can present a major threat to overseas and undersea vehicles. The countering of a swarm of UUVs is significantly challenging to date.
- Threat to undersea infrastructure. Mischievous UUVs can present a threat to undersea infrastructure (e.g., optical fiber cables [136], and oil/gas pipelines undersea [137]).

### 7.3. Detection, Tracking, and Classification Using Active Sonar and Passive Sonar

Common EM waves cannot travel deep underwater due to high attenuation. Extremely low frequency (ELF) EM waves can be used for deepwater propagation [138]. However, ELF requires extremely large antennas. Compared to EM waves, the sound waves (acoustic signals) travel deeper and faster underwater. In addition, the intensity of the sound waves is higher underwater than in the air. Therefore, sound waves are well suited for underwater propagation [139]. However, compared to EM waves in the air, the speed of sound is not constant underwater. The speed and range of sound waves depend on the pressure, salinity, and temperature under the water.

Moreover, a thermocline layer is formed under seawater due to changes in water temperatures at certain depths [140]. The thermocline layer can affect sound wave signaling. In general, if the sound waves emitter/receiver and underwater vehicle are above and below the thermocline layer, then the probability of detecting the underwater vehicle is significantly low due to refraction (bending) of the sound waves from the thermocline layer. There are multipath sound signals due to refraction from the thermocline layer, refraction at large depths (before the bottom of the sea), reflection from the bottom of the sea, and reflections from different objects underwater. Multipath sound signals underwater make the detection and classification of underwater vehicles difficult.

**Figure 11:**
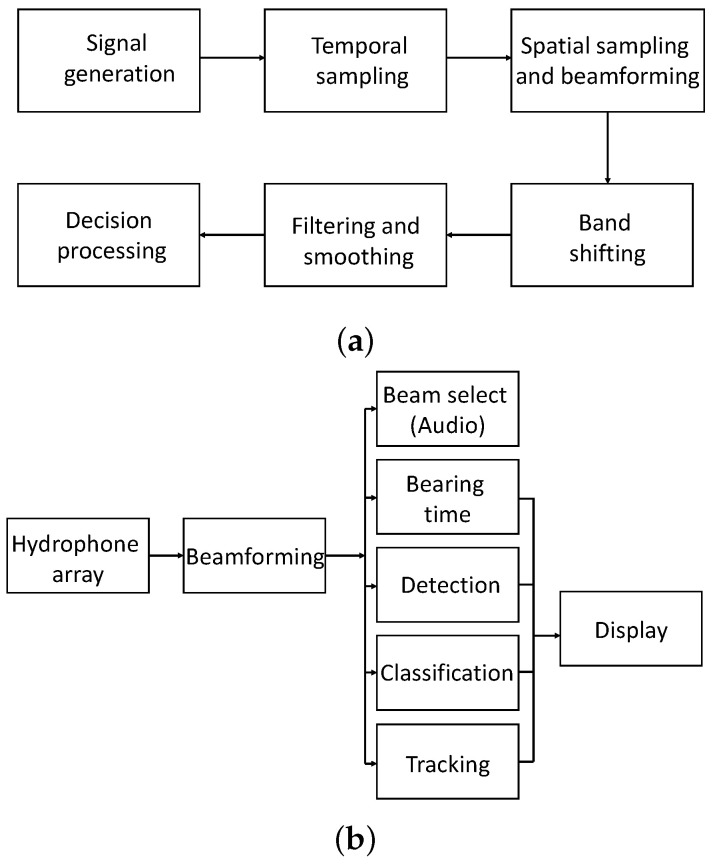
Block diagram of signal processing for (**a**) active sonar, (**b**) passive sonar.

**Figure 12:**
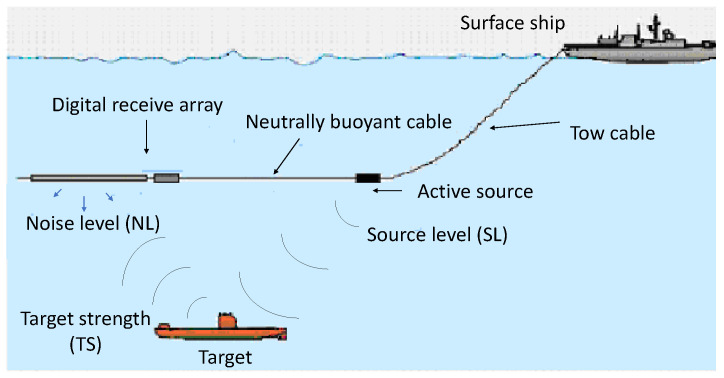
A towed active sonar carried by a surface ship for detection of a submerged UUV/MUV. The sonar is towed to avoid noise (sound) from the carried vessel.

**Figure 13:**
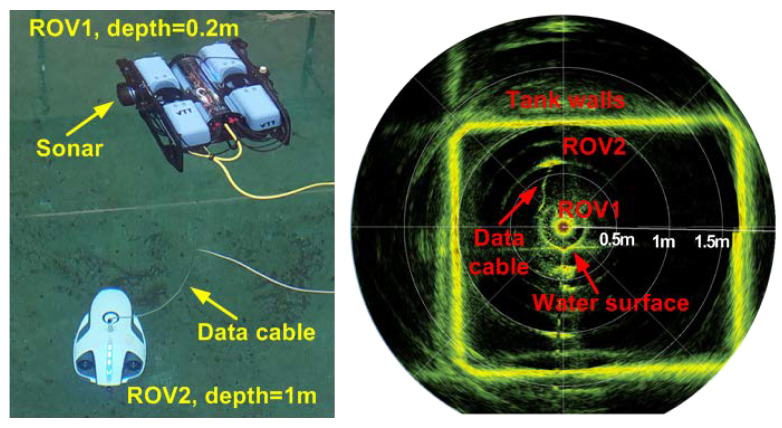
Sonar-based detection of a remotely operated underwater vehicle. Active sonar equipment is mounted on a remotely operated underwater vehicle. Detection is highlighted in yellow in the sonar scan.

Sonar is based on the transmission and reception of sound signals and is used for two major purposes. One is underwater environment mapping and imaging [141], and the other is underwater vehicle detection, tracking, and classification. The detection of an underwater vehicle can be performed using sonar in the active or passive mode [142]. Active sonar sends out particular frequency sound pulses into the water. The reflected sound waves from different objects are collected to determine the presence of an underwater vehicle. A signal processing diagram of active sonar is shown in Figure 11a. The selection of the frequency of the active sonar depends on the requirement. For example, low-frequency sound pulses can be used for long-range detection, whereas high frequency is used for high-resolution imaging. The different frequencies used by sonar and corresponding applications are shown in Table 5. The active sonar equation [143] is given as follows:

(6) $${\left(S/N\right)}_{a}\;\left(dB\right)=SL-2TL-\left(NL-DI\right)+TS,$$

where SL is the source level, TL is the transmission loss, NL is the noise level, DI is the directivity index of the receiver or array gain, and TS is the target strength. An active towed sonar array scenario is shown in Figure 12. An active sonar setup for the detection of UUV is shown in Figure 13, where the sonar is mounted on a remotely operated vehicle (ROV) or UUV. Active sonar is used to detect another ROV underwater. Imaging of the measurement environment is also obtained using the sonar in Figure 13.

A major limitation of active sonar is counter-detection [144]. Counter-detection is the detection of the originating source. Compared to active sonar, passive sonar only listens to the sound/noise from surrounding objects. A block diagram of signal processing using passive sonar is shown in Figure 11b. Differentiation of the sound of an underwater vehicle from the surroundings is a challenging task for passive sonar systems. Spectral analysis of the sounds/noise from various objects in the surroundings is generally carried out. The spectral analysis and a training database of possible underwater objects are used for underwater vehicle identification by passive sonar. The performance of the passive sonar depends mainly on the noise floor of the underwater environment. For example, near busy shipping lanes or fishing areas, the environment is noisy and cluttered; therefore, the detection of underwater vehicles becomes challenging. A possible solution to improve the detection performance in a noisy environment is to use a network of passive sonar. In the case of passive sonar, the target strength TS in (6) will be absent, so the equation will be as follows:

(7) $${\left(S/N\right)}_{p}\;\left(dB\right)=SL-TL-\left(NL-DI\right).$$

The conventional detection, tracking, and classification methods using sonar are currently not able to cover a wide spectrum of modern underwater threats. The conventional techniques to detect and track MUVs using sonar [145,146] may not be effective for UUVs, and new innovative techniques are required. In [147], narrowband and broadband acoustic passive signal processing is used for the detection, localization, and tracking of UUVs. In [148], an active non-imaging sonar is used for underwater detection and localization, which could be used for UUV. The active sonar in [148] uses consecutive transmission of reference and spiral signals. Bearing, range, radial velocities, and tracking of the underwater objects were also carried out in [148]. The classification of underwater objects is carried out using a convolutional neural network applied on sonar images in [149]. The classification is applied on sonar images obtained through a platform mounted on a UUV. The results show that deep learning provides better performance compared to other feature extraction methods in [149].

Target motion analysis (TMA) is a popular method for passive sonar systems to find and track underwater objects [150]. TMA provides the position, course, and speed of an underwater moving object. TMA uses standard geometric techniques for location estimation. However, TMA has limitations in simultaneously detecting multiple objects that are moving randomly (e.g., UUVs at different depths). Another popular approach for finding the location of an underwater moving object is by using an array of hydrophones [151]. The array of hydrophones can be mounted on the surface of MUVs or UUVs or towed away from the surface by underwater vehicles [152]. The spatially spaced array elements are used to capture sounds from different directions to find the location of the desired object. The hydrophone arrays can be one dimensional, two dimensional, or three dimensional. A one-dimensional array can only provide the range information, whereas azimuth angle information can be obtained by a two-dimensional array. A three-dimensional array can provide both azimuth angle information and the depth of the desired object underwater.

### 7.4. Future UUV Uses and Directions for Countering Malicious UUVs

There are many potential uses of UUVs in the future. In particular, autonomous UUVs are expected to dominate major future applications at sea. Major applications of UUVs include the following:

- High-resolution underwater mapping of the seafloor, sea condition monitoring in a given area, and predicting turbulence and other unexpected changes in the water, similar to a weather forecast.
- Monitoring of marine flora and fauna.
- Navigation system similar to GPS using UUVs underwater.
- UUVs can be used for search and rescue and to help in disaster management. UUVs can also be used by law enforcement departments at sea.
- UUVs on the sea surface can provide radio beacons that can assist in long-distance communications in case the satellite link is down/not available. The radio beacons from UUVs can ensure that the ships move in designated lines and avoid collision.
- An underwater network of sensor nodes using UUVs can be created that can assist in different remote sensing applications.
- Monitoring and maintenance of underwater infrastructure, such as oil rigs and underwater oil and gas pipelines, power generation stations at sea, and optical fiber.

The detection, tracking, and classification of malicious UAVs over oceans can be performed with a network of UUVs. A network of mobile UUV sensor nodes can be established that covers vast areas of the oceans. The mobile sensor nodes can work autonomously to position themselves optimally for data collection and analysis. The UUVs in the sensor network can be assigned different tasks. For example, one group of UUVs can be assigned the task of communications with the infrastructure above the water, while another group of UUVs in the network can be assigned the task of positioning reference, and other members of the network can be equipped with hydrophones for detection and tracking.

Distributed sensors (e.g., submerged tethered hydrophones) can be installed on oil rigs, large cargo ships, fishing vessels, and so on. The distributed sensors can help to detect suspicious UUVs in the vicinity. The data from the distributed sensors can be relayed via satellite links to a central point for analysis. Satellite imaging specifically focused on detecting and tracking malicious UUVs when they surface over water can also be used. Furthermore, cognitive acoustic and software-defined sonar networks can be used for the detection and tracking of UUVs. The cognitive adjustment of sonar parameters can increase detection and tracking accuracy against malicious UUVs.

## 8. Conclusions

The challenges posed by UAVs and UUVs have significantly increased in the recent decade. However, there are limited capabilities at present to defend against these challenges. In this study, we have covered some of the key features and capabilities of UAVs and UUVs; the threats and challenges from UAVs and UUVs; and different detection, tracking, and classification methods currently available against malicious UAVs and UUVs. The limitations of the current methods and future directions for detection, tracking, and classification of UAVs and UUVs are also provided.

## Figures and Tables

**Table 1:** Popular categories of UAVs, major types of sensors installed, and their functionalities and features are provided. All the UAVs are equipped with GPS receivers, internal navigation unit(s), and control and data communication links.

UAV Name	Category	Powered by and Sensors Installed	Functionalities	Maximum Payload, Speed, Flight Altitude and Duration
Malat Mosquito	Micro/nano, fixed-wing	Battery, camera, EO sensor	Surveillance and reconnaissance	0.25 kg, 13 m/s, 0.15 km, 1 h
Aurora Skate	Micro/nano, fixed-wing	Articulating motor pods, camera, EO/IR sensors	Surveillance and reconnaissance, tracking objects	0.2 kg, 25 m/s, 4.3 km, 1 h
CyberQuad mini	Micro/nano, multi-rotor	Battery, camera, and multiple sensors for detecting gases and pollutants	Urban aerial reconnaissance, detection of gases, industrial and other pollutants	1.5 kg, 18 m/s, -, 0.67 h
RQ-11 Raven	Small/mini, fixed-wing	Battery, camera, infrared sensor, miscellaneous sensors	Surveillance, mapping, imaging and object detection and classification	0.2 kg, 22.5 m/s, 4.2 km, 1.5 h
Matrice-600	Small/mini, multi-rotor	Battery, camera, intelligent batteries	Imaging and surveillance, high data rate live streaming	6 kg, 18 m/s, 2.5 km, 0.67 h
SkyEye Sierra VTOL	Small/mini, fixed-wing and multi-rotor	Battery/petrol engine, camera, surveying and surveillance equipment, multiple sensors	Imaging, mapping, inspection, and surveillance, and other sensing applications	3 kg, 30 m/s, 3 km, 5 h
Watchkeeper	Medium, fixed-wing	Rotary Wankel engine, camera, EO/IR sensor, motion filter	Imaging, surveillance and reconnaissance	150 kg, 40 m/s, 4.9 km, 14 h
Eagle Eye, Bell HV-911	Medium, tiltrotor	Turboshaft engine, camera, surveillance sensors, rescue equipment	Search and rescue, surveillance, reconnaissance (mainly at sea)	90 kg, 103 m/s, 6 km, 5.5 h
Skyeye-R4E	Medium, fixed-wing	Twin rotor rotary engine, camera, surveillance and miscellaneous sensors	Imaging, surveillance, pesticide spraying, border patrols	82 kg, 55 m/s, 4.6 km, 8 h
Global Hawk	Large, fixed-wing	Turbofan engine, camera, EO/IR sensors, laser and radar warning receivers, ECM equipment, MTI system	Long endurance and high-altitude and wide area ground/sea surveillance and reconnaissance, communications	1400 kg, 175 m/s, 18 km, 33 h
Zephyr 8	Large, fixed-wing	Solar-powered, Amprius lithium-ion batteries, communication systems	Airborne communications: as a mobile communication relay	5 kg, 9.5 m/s, 21.3 km, 26 days

**Table 2:** Comparison of current and expected future UAV features and capabilities.

Serial #	Current UAV Features and Capabilities	Future UAV Features and Capabilities
1	Multi-rotor or fixed-wing	Hybrid of multi-rotor and fixed-wing, variable wing geometry
2	Aerial flying	Aerial, over- and underwater, and on-ground maneuvering
3	Propeller propulsion	Jet engine propulsion in addition to propeller propulsion
4	Battery and fossil fuel	Solar, synthetic, hydrogen fuel, and battery charging while flying
5	Small and medium payloads	Large, multi-purpose payloads
6	Limited maneuverability for fixed-wing UAVs	High maneuverability for fixed-wing UAVs
7	Cost varies, and dependent on the size of UAV	Reduction in price of different sizes of UAVs
8	Flight duration dependent on the payload	Long flight duration, and less dependent on the payload
9	Weather and light affects performance	All weather, day and night high performance
10	Small to medium RCS	Very small RCS
11	Limited and vulnerable communication links	Redundant and secured communication links
12	Semi-autonomous operations	Fully autonomous and AI-controlled options available
13	Limited ECM capabilities	Enhanced ECM capabilities

**Table 3:** Comparison of current and future research directions for countering malicious UAVs.

Current Countermeasures Cgainst UAVs	Future Cirections of Countermeasures against UAVs
Radars Acoustic analysis RF analysis EO/IR imaging RF jamming, remote hacking, and GPS spoofing High-power laser and EM radiation Nets and prey birds	Modifications to current radar systems specifically for UAVs Mesh of laser beams Distributed sensors at different locations AI-aided cognitive analysis of the threat using multiple sensors Regulating legitimate UAV aerial traffic through tagging Use of satellite networks for UAV detection and tracking

**Table 4:** Popular UUVs, types of sensors installed, dimensions of the UUVs, and maximum endurance and depths underwater are provided. All the UUVs provided here are autonomous and are used for deepwater and seafloor imaging/mapping and oceanography research.

Sr. #	UUAV Name	Powered by and Sensors Installed	Dimensions, Endurance and Depth
1	MBARI’s Dorado-class	Battery, 200 kHz multibeam sonar, 100 kHz and 410 kHz chirp sidescan sonars	0.5 m diameter, 6.4 m length, 20 h endurance, 6 km depth
2	Sentry	Battery, conductivity, temperature and depth sensors, digital camera, reodx potential probe	1.8 m height, 2.2 m width, 2.9 m length, 24 h endurance, 6 km depth
3	Qianlong-1	Battery, camera, obstacle avoidance sonar, side-scan sonar	0.8 m diameter, 4.6 m length, 24 h endurance, 6 km depth
4	SeaBED Class	Battery, Imagenex Delta-T imaging sonar, camera	2 m length, 1.5 m height, 1.2 m width, 24 h endurance, depth 5 km
5	Urashima (hybrid)	Battery, multi-beam echo sounder, Niskin water sampler, interferometric synthetic aperture sonar, gravimeter system	1.3 m width, 10 m length, 24 h endurance, depth 3.5 km
6	Aster x/Idef x	Battery, multi-beam echo sounders, multiple sensors, sub-bottom profilers, spectrometers	0.7 m diameter, 4.5 m length, 16 h endurance, depth 3 km
7	BlueROV2	Battery, gyroscope, accelerometer, magnetometer, pressure/depth and temperature sensor	457 mm × 338 mm × 254 mm, 4.5 m, 4 h endurance, depth 300 m

**Table 5:** Different frequencies and applications of sonar.

Serial #	Frequencies	Application
1	3–30 Hz, ELF band	Underwater communications and pinging
2	30–300 Hz, super low frequency (SLF) band	Underwater submarine communications
3	300 Hz–3 kHz, ultra low frequency (ULF) band	Underwater communications through dirt and rocks
4	3 kHz–30 kHz, very low frequency (VLF) band	Near-sea-surface communications, navigation beacons
5	30 kHz–300 kHz, low frequency (LF) band	Near-sea-surface communications, navigation beacons
6	3 kHz–30 kHz, VLF band	Near sea surface communications, navigation beacons
7	Less than 1 kHz, 1 kHz–10 kHz, greater than 10 kHz, and less than 30 kHz	Active sonar operation
8	50 kHz, 120 kHz, 200 kHz, and 455 kHz	Sport fishing
9	120 kHz, and 200 kHz	Sea floor imaging using deep towed sonar, and swath phase-bathymetric mapping
10	100 kHz–1 MHz	Side-scan sonar
